# Management of Aggressive Behavior in Emergency General Settings: Specialized Practice in Mental Health Nursing

**DOI:** 10.1192/j.eurpsy.2026.11516

**Published:** 2026-07-31

**Authors:** P. Pires, C. Laranjeira

**Affiliations:** 1 School of Health Sciences; 2 Centre for Innovative Care and Health Technology, Polytechnic University of Leiria, Leiria, Portugal

## Abstract

**Introduction:**

The challenges facing healthcare settings are varied, relating to safety in care. Nurses’ ability to manage aggressive behavior in a general emergency setting contributes to creating a safer care environment and, consequently, to a culture of safety.

**Objectives:**

To train emergency room nurses in strategies for managing aggressive behavior through specialized intervention in Mental Health and Psychiatric Nursing.

**Methods:**

This was a pre-experimental, single-group study with pre- and post-intervention assessments. Data were collected through a questionnaire with participants’ sociodemographic and professional data; the Kessler Psychological Distress Scale; the Rathus Assertiveness Scale (RAS); the Inventory of Relational Helping Skills (ICRA); and the Attitudinal Connotations of Aggression Scale (ECADA).

**Results:**

A decrease in passivity levels was found post-intervention. Regarding nurses’ attitudes toward aggressive patients, there was an increase in the mean score for communicative connotation, resulting from the user’s feeling of inferiority. Regarding relational helping skills, there was a slight increase in the mean score for communication skills, and the mean score for contact skills remained unchanged. However, no statistically significant differences were identified in any of the variables assessed, pre- and post-intervention.

**Conclusions:**

The program’s implementation highlighted the importance of developing nurses’ relational and communication skills in managing aggressive behavior. It is suggested that there is a need to invest in ongoing training for healthcare professionals in verbal and nonverbal communication skills to prevent communication errors, which could lead to violent acts in healthcare.

**Disclosure of Interest:**

None Declared

